# Lightweight Active Object Retrieval with Weak Classifiers

**DOI:** 10.3390/s18030801

**Published:** 2018-03-07

**Authors:** László Czúni, Metwally Rashad

**Affiliations:** 1 Department of Electrical Engineering and Information Systems, University of Pannonia, Veszprém 8200, Hungary; metwally.rashad@virt.uni-pannon.hu; 2 Department of Information System, Faculty of Computers and Informatics, Benha University, Benha 13518, Egypt

**Keywords:** object retrieval, Hough transformation, sensor fusion, active vision

## Abstract

In the last few years, there has been a steadily growing interest in autonomous vehicles and robotic systems. While many of these agents are expected to have limited resources, these systems should be able to dynamically interact with other objects in their environment. We present an approach where lightweight sensory and processing techniques, requiring very limited memory and processing power, can be successfully applied to the task of object retrieval using sensors of different modalities. We use the Hough framework to fuse optical and orientation information of the different views of the objects. In the presented spatio-temporal perception technique, we apply active vision, where, based on the analysis of initial measurements, the direction of the next view is determined to increase the hit-rate of retrieval. The performance of the proposed methods is shown on three datasets loaded with heavy noise.

## 1. Introduction

The visual surveillance and recognition of 3D objects by unmanned aerial vehicles (UAVs) and by autonomous robots, and the replacement of bar/matrix codes (used for the identification of products) with the objects’ views, are key technologies for future applications. There are many approaches for such problems within computer vision, but the researcher or developer is now concerned with how to balance recognition performance with the cost of the technique (“cost” here refers to computational complexity, memory usage, time of recognition, and time of training). For this reason, there is an increasing need for lightweight sensors and signal processing technology that can collect and process valuable information from their environment [1,2,3] to make proper decisions. To find an optimal solution within an application framework, the recognition (or retrieval in many cases) itself should be considered as an information gathering and decision making process, so active perception should be carried out.

In this study, we set out to solve a “specific object” retrieval problem, where the task is to retrieve given 3-D objects based on their visual appearance while keeping the computational and memory costs low. To achieve a high hit-rate, we continuously processed the images of the video camera while the object was targeted, measured the orientation of the camera, and evaluated the possible candidates with the help of compact visual descriptors. This approach can be implemented in a dynamic recognition model, where even the orientation of new image queries can lead to faster and more accurate recognition.

Clearly, video provides more visual information about 3D objects than a simple 2D projection can. A 3D structure can also be reconstructed from motion techniques. However, these approaches generally require high quality images, camera calibration, and high computational power. Here, we investigated methods where 3D reconstruction is not used, but 2D views from different directions are fused in viewer-centric models.

In cognitive science, there is a debate about how the human brain uses images to represent 3D objects, i.e., whether object- or viewer-centered representations are used [4]. Similarly, in computer vision, there are object-centered representations, which use features (e.g., boundary curves, 3D points, and surfaces) to model objects in space, and there are viewer-centered representations via 2D projections of the appearance of objects as captured from different viewpoints.

In recent years, there has been a significant evolution in computer vision with the involvement of deep neural networks (DNNs), especially with convolutional neural networks (CNNs) for object detection and recognition. These techniques can also be considered as appearance-based approaches and can usually reach high recognition rates in many scenarios. However, it is also clear that they have high computational and memory demands usually solved with the application of GPUs or other parallel programming techniques implemented many times on a remote server. While there is much effort to reduce the amount of resources of DNNs required [5], making them data-driven (i.e., easy to apply to different datasets, easy to adapt, and easy to extend) is also a task to be completed in the future.

Contrary to techniques that are used as an attempt to reconstruct 3D models of objects for recognition, and to techniques that utilize complex DNN models, our purpose (and main contribution to previous results) is to show that weak classifiers, obtained by a sequence of accumulated visual information and an orientation of the camera, can be successfully used to achieve lightweight object retrieval in an active recognition process. The fusion is achieved by the well-known Hough framework, and the selection of a new viewpoint is based on the discriminative ability of the different viewing directions of the most probable candidates. We used 2D camera images with a resolution of 640 × 480, and we used the relative orientation of a low cost orientation sensor available in many mobile devices. To investigate the robustness, we applied heavy additive Gaussian noise and motion blur on the COIL-100 [6], the SMO [7], and the SUP-16 datasets.

Our article is organized into seven sections: In Section 2, we introduce alternative approaches that are closely related to ours. Since there is a large number of such papers, we cannot give a comprehensive overview; rather, we discuss articles that are interesting from a certain point of view. In Section 3, the viewer-centered representation model is explained, and in Section 4 and Section 5, the retrieval methods, named Hough-Based Retrieval (HBR) and Active-Hough-Based Retrieval (AHBR), are described. To evaluate the performance of the proposed techniques, a large number of experiments are performed. Descriptions and evaluations of these experiments are in Section 6, while a discussion and conclusions can be found in Section 7.

## 2. Related Papers

Object recognition and retrieval are very broad topics in computer vision and pattern recognition. Since the possibilities of the application of such systems are countless there is a larger interest in the research of the different aspects of optical object recognition [8]. Note that, while there is difference between the meaning of the terms “recognition” and “retrieval,” we can use both in our framework. In this article, we will technically present retrieval (where the object to be recognized is previously trained and we choose the best candidate), but recognition (where untrained objects can also be queried) can also be achieved by analysis of the confidence of the best candidate ending in rejection if necessary. Please see [9] for such extension.

Thus, in this article, we cannot give a general overview. We focus on specific papers that try to solve similar problems with congenial approaches. A good general overview about visual object recognition can be found in [10], and a DNN-specific overview can be found in [11]. There are two main topics that are closely related to our approach: video-based or multi-view object recognition and active vision, which is often found in robotics-related journals and conferences.

Multi-view- and video-based recognition papers have appeared very early; for example, the authors of [12] focused on the problem of large data sizes of 3D recognition. They introduced a so-called parametric eigenspace method: the compact representation of 3D object appearance was parameterized by pose and illumination. The large image set, obtained by automatically varying pose and illumination, was compressed to obtain a low-dimensional subspace, called the eigenspace, in which the object is represented as a manifold. Given an unknown input image, the recognition system projected the image to eigenspace and the object was recognized based on the manifold it lies on. The exact position of the projection on the manifold determined the object’s pose.

In [13], recognition was achieved from video sequences by employing a multiple hypothesis approach. Appearance similarity and pose transition smoothness constraints were used to estimate the probability of the measurement being generated from a certain model hypothesis at each time instant. A smooth gradient direction feature was used to represent the appearance of objects, while the pose was modeled as a von Mises–Fisher distribution. Recognition was achieved by choosing the hypothesis set that had accumulated the most evidence at the end of the sequence. Unfortunately, the testing of the method was carried out on four objects only.

In [14], the underlying topological structure of an image dataset was generated as a neighborhood graph of features. Motion continuity in the query video was exploited to demonstrate that the results obtained using a video, compared to results obtained using a single image, are more robust albeit more intensive computationally.

There are approaches where different types of visual features are extracted and utilized to increase the performance of recognition. In [15], textural and shape features are used as complements of each other for video-based object recognition. Their relative weighting is learned by optimizing discriminative performance on synthetically distorted data. To use different visual features, the bag of visual words (BoVW) approach is often used in computer vision. For example, in [16], the BoVW is used for object recognition while solving the problem of eliminating foreground and background features: highly salient patches have an important role in describing an object, while those with low saliency, instead of discarding them, are still taken into account albeit with low emphasis.

While the above approaches involve only 2D images, in [17], an accelerometer and a magnetic sensor was used to extend visual information to recognize landscapes. Clustered SURF features for visual words-based classification and for tracking a FAST corner detector was used in a server–client environment. Similarly, in [7], the proposed methods, using SIFT points of tracked objects rotated in front of a camera, were used and about an 80% accuracy was achieved, but the complexity needed for implementation directly on mobile platforms was too high.

Active recognition is also a relatively old idea in pattern recognition, and non-active methods have been extended many times. For example, the work [18] can be considered as an extension of [12] (described above) in that a viewing position that minimizes the average entropy was chosen. Without mentioning many of such techniques, we refer to the survey in [19] and shortly discuss a few new results which aim at lightweight recognition.

In [20], hypotheses about objects in the hand of an iCub robot were created and updated as the recognition progressed. They adopted probabilistic Monte Carlo localization methods to maintain a high number of hypotheses in parallel, and they used particle filtering, regarding hypotheses, to take into account the viewpoint changes in the form of proprioceptive information obtained from the robot arm. Unfortunately, the tests included only six real objects, so a real-life evaluation of the proposed strategy was not presented.

The method used in [21] used SIFT features for active object recognition and verification. They created an automatic viewpoint selector that uses a vocabulary tree structure to weigh the uniqueness of each feature in a viewpoint. Every viewpoint was then given a value that was obtained by summing the uniqueness measure of all its features. This mechanism is used to select the subsequent view. While the experiments, with only a few test objects, showed that the view selection mechanism is successful, the running time and other practical questions (the effects of noise, the estimation of orientation, and cases with large datasets) were not discussed.

In [22], a method for active recognition using an RGB-D (Red Green Blue - Depth)camera mounted on a quadrocopter, using an object bounding box, color, SIFT, and a viewpoint feature histogram for recognition, is discussed. The authors define a utility score for a particular action, which can be computed by mutual information (MI). MI is used to reduce the uncertainty of the current object’s class and its pose if a new observation is made. If a prior map of the environment is given, the change of the object class or the change of its orientation can be detected.

By contrast, our model works with relative orientation since the object can be moved and rotated. Additionally, our approach is designed to be lightweight and thus uses compact visual descriptors and the scalar orientation data often available from low-cost IMU (Inertial Measurement Unit) sensors. Noise tolerance and efficiency was found to be achieved with a series of observations (rather than with deeper processing of complex data). New viewpoints could be chosen in a way to serve with discriminative data. Our first attempts to fuse optical and orientation information for object retrieval have been documented in [9,23]. The main contribution of this article, besides the analysis of the contribution of orientation information in the Hough framework, consists in showing that the proposed technique can be used for active perception and thus that very lightweight techniques can be efficiently used for 3D object retrieval.

## 3. Viewer-Centered Visual Representation with Orientation Data

While, in many applications, the purpose is to achieve general automated surveillance or simultaneous localization and mapping (SLAM) by robots [22], we here propose a solution for the visual recognition of specific 3D objects that can be seen from various directions. The reliability of classification is supported by multiple observations and continuous (but lightweight) data processing. This approach is straightforward since, in real-life applications, single viewpoints may be of poor quality and simply may not contain sufficient information to reliably recognize objects. A further complication arises if two or more objects have similar views with respect to a feature set. In addition, it is well-known that the appearance of objects depends on several factors such as the viewing geometry, illumination, and the presence of noise increases the difficulty.

The use of multiple views from different viewpoints can make the 3D object recognition problem more tractable and scalable. If the observed features can be processed fast, then we can construct classifiers and fuse their decisions. This kind of combination of weak classifiers is known to be robust in many pattern recognition tasks [24]. If orientation information can be included in the fusion process, then, if the processing speed allows, active perception can also be achieved as shown in our paper.

To construct an efficient technique, the image feature descriptors, the retrieval mechanism, the storing database, and the feature similarity measure should be carefully designed to minimize the amount of data space and the retrieval time and to maximize the hit-rate. While, to obtain a complete object model, several azimuth and elevation angles are required, for average applications, the variations in elevation can be limited (in our tests, we used only one elevation angle typical for an object placed on table).

In [25], it is shown that the color and edge directivity descriptor (CEDD) [26] is a robust visual tool and can be computed quickly in mobile platforms. The CEDD is an area-based descriptor where pixels are classified into one of six texture classes (non-edge, vertical, horizontal, 45 and 135 degree diagonal, and non-directional edges) with the help of the MPEG-7 Edge Histogram Descriptor. For each texture class, a (normalized and quantized) 24 bin color histogram is generated, and each bin represents colors obtained by the division of the HSV color space. Other types of descriptors can also be used with different advantages and disadvantages. Without going into the investigation of the optimal descriptor, there is no doubt that the size of the CEDD is very attractive for lightweight techniques: feature vectors with a length of 144 (6 × 24) will serve as the input of weak classifiers. Note that a single point SIFT descriptor is of size 128. The similarity of CEDD vectors is computed with the Tanimoto coefficient [26]:

(1) $$T\left(q_{i},c_{j}\right)=\frac{d{\left(q_{i}\right)}^{T}d\left(c_{j}\right)}{d{\left(q_{i}\right)}^{T}d\left(q_{i}\right)+d{\left(c_{j}\right)}^{T}d\left(c_{j}\right)-d{\left(q_{i}\right)}^{T}d\left(c_{j}\right)}$$

where $d{\left(q_{i}\right)}^{T}$ is the transposed vector of the descriptor of a query (and the same goes for a candidate $c_{j}$). Rotational invariance can be achieved as given in [23].

To include the viewing direction into our model, we need to know the relative orientation of the camera. Low-cost IMU sensors can serve with reliable orientation information for our purposes [27]. Visual observations will be processed sequentially, where, besides the visual descriptors, the difference of the orientations of the camera will be evaluated.

As we have to run several queries in our multi-view approach, fast searching mechanisms are required. The KD-Tree is an efficient data structure published in [28]. Since it is often used for indexing and retrieval, in [29], the authors improved it to index a large number of SIFT and other types of image descriptors. They also extended priority search among multiple trees simultaneously. In our case, a single KD-Tree, containing the CEDD descriptors of all objects, was used, as the number of candidate views (typically below 100,000) does not require the use of such multiple tree solutions. The tree was built in a standard way, based on the variance and mean of CEDD descriptors, as given by [28].

## 4. Combining Weak Classifiers and Fusion with Orientation Data in the Hough Paradigm

The Hough transform was originally used for the identification of lines. Later, it was extended to identify the positions of other shapes like circles and ellipses. It is also known to have been efficiently used for object detection [30] and action recognition [31]. In our approaches (HBR and AHBR), we used this paradigm to combine weak classifiers to obtain a strong retrieval mechanism. There are three main steps of retrieval:

1. Observation and feature extraction: camera images are processed to generate (CEDD) descriptors ($d_{i}$); in the meantime, we record the orientation of the camera ($o_{i}$) at the actual viewpoint.
2. Voting: each occurrence of a descriptor and the belonging orientation information vote for a candidate $c_{j}$ with weight $\theta (c_{j},d_{i},o_{i})$.
3. The Hough Score for a candidate is given:
  (2) $$H\left(c_{j}\right)=\sum_{i}\theta \left(c_{j},d_{i},o_{i}\right),$$

and the retrieval is done by selecting the object with the highest score:

(3) $$\hat{c}=\arg\max_{i}H\left(c_{i}\right).$$

Orientation data cannot be used as part of the descriptors; rather, the difference between the orientation of two views will be used: orientation is relative, and the object can be rotated in its position any time.

**Figure 1:**
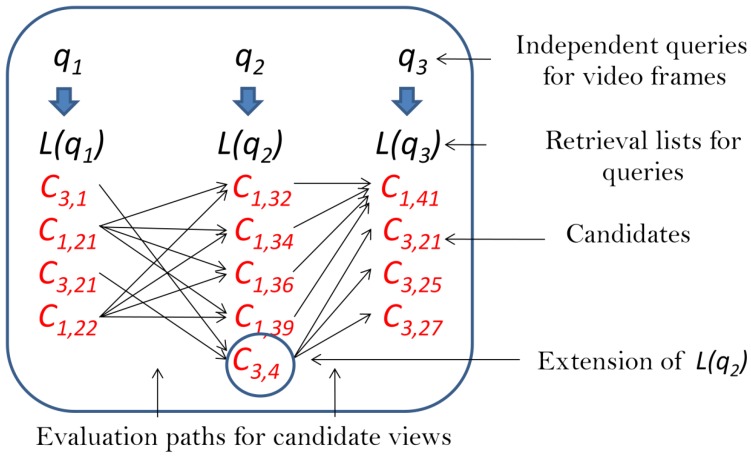
Possible paths defined by candidate objects on the retrieval lists ($N^{q}=3$) to evaluate the Hough score by Equation (4). Note that object $c_{3}$ was not on $L(q_{2})$ initially, so one of its views was added to be able to obtain its score. Different strategies for this step are discussed in Section 6.2.

Any object model (composed of $N^{M}$ views and the corresponding orientations) can give a candidate for a given query image. Since the appearance is compactly represented with CEDDs, a query can be answered in a very short time via KD-Tree indexing. To limit the number of voting candidates, all $q_{i}$ queries ($N^{q}$ frames of the video sequence) generate their own retrieval list $L(q_{i})$ of limited length (typically between 4 and 10) and with $c_{j,k}$ elements (object *j*, view *k*) by running independent searches in the object models. The significance of maintaining short lists, containing only a few candidates with the highest votes (highest Tanimoto coefficients), is that we should avoid extensive searches rather than assume that the right candidate will appear on at least one of the lists.Thus, we have a sequence of short retrieval lists, one for each query, and all the retrieved candidates on the lists provide votes based on visual similarity and relative orientation:

(4) $$\begin{array}{cc} H(c_{j})= & \max_{c_{j,k}\in L\left(q_{i}\right)}[\sum_{i=1}^{N^{q}}T\left(q_{i},c_{j,k}\right)-\omega \sum_{i=1}^{N^{q}-1}|o_{\Delta }\left(q_{i}\right)-o_{\Delta }\left(c_{j,k}\right)\left|\right] \end{array}$$

where ω weights the role of orientation, $o_{\Delta }\left(q_{i}\right)$ is the difference of orientation of $q_{i}$ and $q_{i+1}$, and $o_{\Delta }\left(c_{j,k}\right)$ is the same for $c_{j,k}$ accordingly, i.e., $o_{\Delta }\left(c_{j,k}\right)=o\left(c_{j,k}\right)-o\left(c_{j,l}\right)$ where $c_{j,l}\in L\left(q_{i+1}\right)$. Satisfying Equation (3) means the evaluation of Equation (4) by traveling through all paths, connecting the views of the same objects on the retrieval lists (for illustration, see Figure 1). If an object is not on any of the lists, then it is simply out of focus in our search. However, if it appears on any but is missing from some, then those lists are extended with a view of the object. Different strategies to extend the lists are discussed in Section 6.2.

Please note that finding $\hat{c}$ also means estimating the orientation of the corresponding views. In normal cases, the sampling process (the movement of the camera) can be arbitrary and often accidental. However, since we implement retrieval, we have a preconception of possible objects and can plan the sampling to help the discrimination by choosing certain views.

## 5. Active Retrieval to Minimize Ambiguity

Active vision systems can be classified, according to their next view planning strategy, into two groups:

1. systems that take the next view to minimize an ambiguity function;
2. systems incorporating explicit planning algorithms.

We have chosen the first strategy and here introduce a method that is very close to the way in which humans would naturally move around an object to become acquainted with its appearance from different directions to be able to recognize it. Based on a rapid evaluation of the first observations, they hypothesize which objects have high probability to appearand plan their movement to find those views that can reduce ambiguity.

Based on the preliminary models, the $N^{\tilde{M}}$ average views of object *i* from the descriptors is computed within a viewing range (each containing $N^{k}$ views):

(5) $$d\left({\tilde{c}}_{i,k}\right)=1/N^{k}\sum_{l=1}^{N^{k}}d\left(c_{i,l}\right).$$

It is important that $N^{\tilde{M}}<<N^{M}$, which means that each object is now represented by only a few views, computed as average CEDD vectors that equally divide the circle into $N^{\tilde{M}}$ parts. This is necessary to reduce the amount of computations when comparing the different candidates for next view planning. Afterward, the similarity between these average views can be computed with the Tanimoto coefficient (Equation (1)) and can be stored in matrix *S* of size $N^{\tilde{M}}N^{c}\times N^{\tilde{M}}N^{c}$, where $N^{c}$ gives the number of all possible objects.

After making the very first observations, we are to evaluate the retrieval lists $L(q_{i})$ just as described above in Section 4. As $o(c_{j,k})$ provides the estimate of orientation for all $c_{j,k}\in L\left(q_{i}\right)$, we can also compute the similarity of views to the left (and to the right accordingly):

(6) $$S_{left}=\sum_{c_{j},c_{l}\in L\left(q_{i}\right),j\neq l}T\left({\tilde{c}}_{j,left},{\tilde{c}}_{l,left}\right)$$

where ${\tilde{c}}_{j,left}$ is the closest ${\tilde{c}}_{j}$ view left of $o(c_{j})$.

Finally, we should move the sensor either to the left or to the right depending on the dissimilarity of views of the possible candidates:

(7) $$Decision=\left\{\begin{array}{cc} \mathrm{Move}\;\mathrm{to}\;\mathrm{left} & \mathrm{if}\;S_{left}\leq S_{right}\\ \mathrm{Move}\;\mathrm{to}\;\mathrm{right} & \mathrm{if}\;S_{left}>S_{right} \end{array}\right..$$

In our article, we call this approach Active-Hough-Based Retrieval (AHBR). Its performance will be compared to HBR in the next section.

## 6. Experiments and Analysis of Results

The purpose of this section is to show results related to the hit-rate and running times of non-active and active retrieval in the proposed framework. Everything was implemented on a tablet computer equipped with an ARM Cortex A9 processor, and the running time is the average of several such measurements. To be able to collect large amounts of statistical data, we used three datasets and ran systematic tests, during which more than 8000 queries were evaluated. Our experiments ran on a mobile platform, and in the case of the standard datasets, we did not physically move our camera around the objects; rather, we used heavy image noise to simulate the conditions of real life. The inaccuracy of the orientation sensor was measured by repeated experiments, and a random bias was then added to each query based on the measured distribution. In the case of our own small dataset (SUP-16), the images and orientation were recorded simultaneously with the built in camera and orientation sensor.

There are many types of noise that affect the quality of real-life digital images recorded with a moving camera. We used motion blur [32] and additive Gaussian noise [33] on the test images to simulate such scenarios, where observation conditions are not ideal and there is a real need for multiple observations and active vision.

### 6.1. Datasets

The COIL-100 dataset includes 100 different objects with 72 images of each taken at pose intervals of $5^{\circ }$. Since each object was tested 10 times with 8 queries $\left(N^{q}=8\right)$, the query dataset is composed of 10×8 randomly selected images of each object. The SMO dataset, chosen for comparison with the method of [7], contains 25 different objects, where 36 images of each were recorded by rotating them in the plane at $10^{\circ }$ steps. SUP-16 is a small dataset including 16 objects, where 44–73 views per object were captured by our tablet from the same elevation but from different azimuths, leading to approximately 900 images.

**Figure 2:**
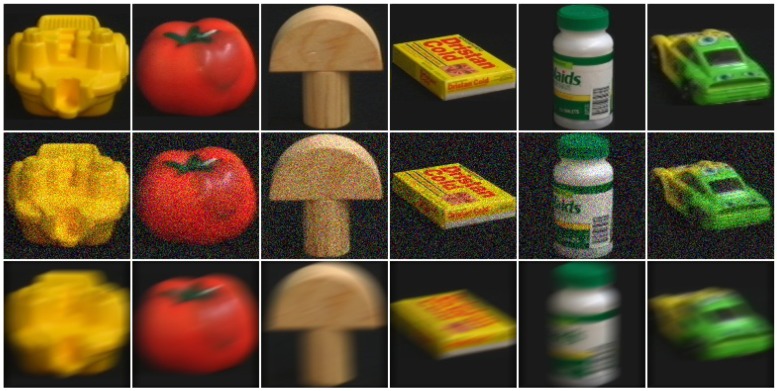
Test image examples from COIL-100 without noise (**first** line), with additive Gaussian noise (**middle** line), and with strong motion blur (**bottom** line).

**Figure 3:**
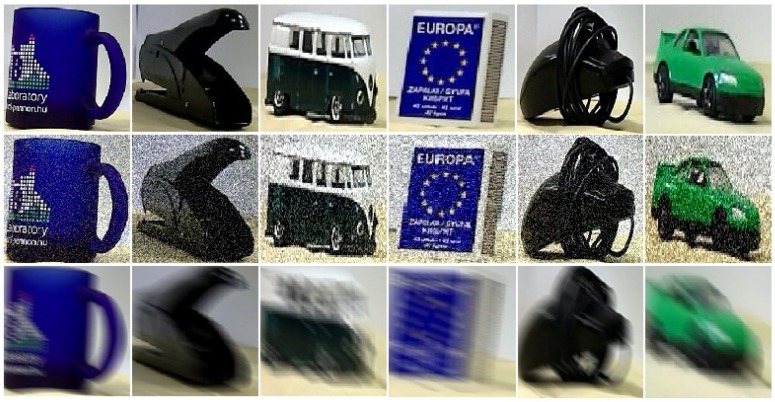
Test image examples from SUP-16 without noise (**first** line), with additive Gaussian noise (**middle** line), and with strong motion blur (**bottom** line).

To have realistic tests, we degraded the distortion-free (DF) query images either with additive Gaussian noise (GN) or with motion blur (MB) (typically occurring when the image is taken under low lighting conditions with a moving camera). For the former, the imnoise function of MATLAB with a standard deviation of 0.012 was used, while for the latter we used the fspecial function with parameters len=15 and angle θ=20 degrees. Some examples of the distorted queries of COIL-100 are shown in Figure 2, while examples of those of SUP-16 are in Figure 3. For testing, we removed the query images and the neighboring image frames (within $10^{\circ }$) from the models, so the closest orientation angle between a query and an available model view was between 10 and $30^{\circ }$, resulting in reduced model sizes (78% of the original for the SMO, and 79% for the COIL-100) and more difficult retrieval tasks.

**Figure 4:**
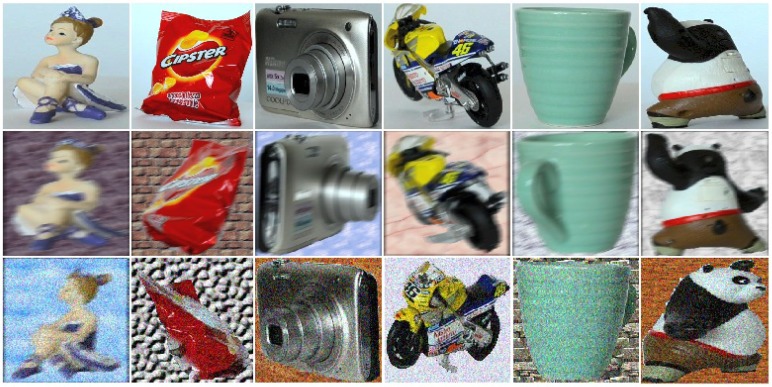
Test image examples from SMO without noise (**first** line), with motion blur (**second** line), and with additive Gaussian noise (**third** line). Various textured backgrounds were added.

To obtain an even more realistic picture of the performance, we ran different evaluations over the SMO dataset. Since the queries of the tests of [7] were not available, we provide only rough quantitative comparisons of the two approaches (HBR and the one in [7]). In order to obtain similar queries as in [7], the 10 randomly selected query images of each object were modified to have different uniform backgrounds (UBs) and different textured backgrounds (TBs) (see Figure 4 for illustration).

### 6.2. The Role of Orientation

One might think that, since there are many rotationally invariant objects, the role of orientation can be insignificant. In this section, we show that, as we increase the role of the orientation information in our model, the hit-rate continuously increases, while the next subsection shows the case where active perception is applied.

**Figure 5:**
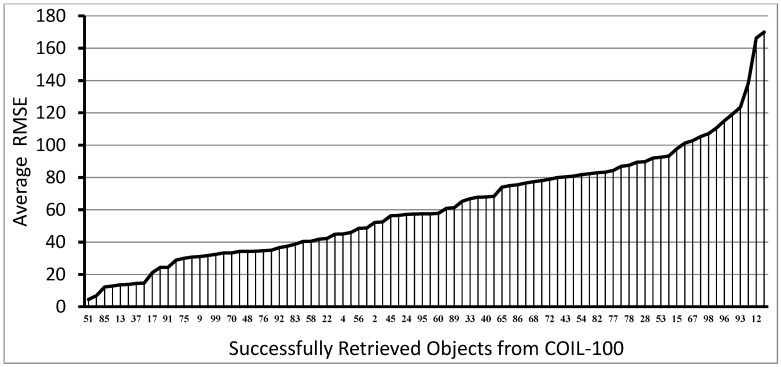
Average root mean square error (RMSE) of the estimated orientation angle of (motion-blurred) objects, at least once successfully retrieved from a trial of 10, with HBR ($N^{q}=2$, ω=0.5).

Figure 5 shows the root mean square error (RMSE) (the average of several experiments) of the estimated orientation angles for all those objects of the COIL-100, when the retrieval was successful with the HBR method. Some objects have quite a large RMSE, while in other cases this error is close to the noise of the IMU sensor. Note that these data are the average of maximum 10 measurements, since not all retrievals were successful for the different objects. The following experimental results show that the proposed retrieval mechanisms (HBR and AHBR) still benefit from orientation information.

When measuring the hit-rate, we compared cases with different roles of orientation:

1. The case where ω=0 in Equation (4) and the insertion of missing candidates to the retrieval lists is based on their visual similarity given by Equation (1) (denoted the HBR Best Visual Match);
2. The case where ω=0 in Equation (4) and the insertion of missing candidates to the retrieval lists is based on their relative orientation (denoted HBR ω=0);
3. The same as above but ω=0.5 (denoted HBR ω=0.5).

**Figure 6:**
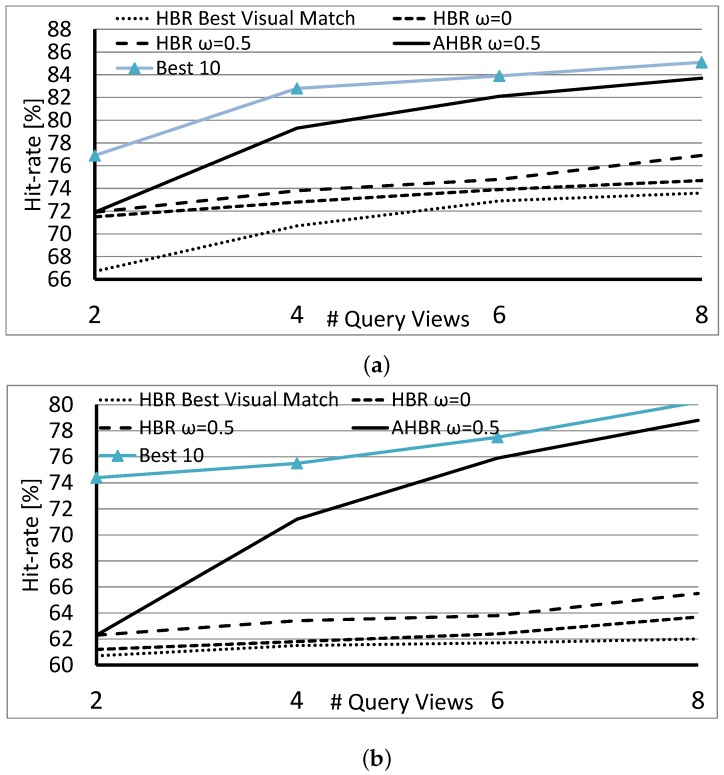
Average hit-rate obtained over the COIL-100 dataset with the HBR Best Visual Match, the HBR ω=0.0, ω=0.5, the AHBR ω=0.5, and with the “Best 10”: (**a**) queries with motion blur (**top)**; (**b**) queries with additive Gaussian noise (**bottom**).

In the first case, the approach is a multi-view recognition technique where the orientation of queries is not exploited at all. When an object appears on any retrieval list but is missing from some, then those lists are extended with a view of the object that has the largest Tanimoto coefficient. As shown in Figure 6, this results in the lowest hit-rates. In the second and third cases, we chose the candidate for extension, which is at the right orientation. In other words, its relative orientation matches the relative orientation of the actual query (that is $|o_{\Delta }\left(q_{i}\right)-o_{\Delta }\left(c_{j,k}\right)|$ is minimized). After experiments with the settings of the weight of the orientation term, we found that ω=0.5 yielded the best results in general.

**Figure 7:**
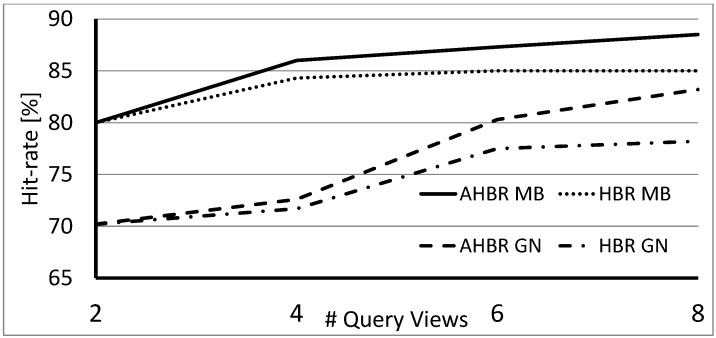
Average hit-rate obtained over the SUP-16 dataset with HBR and with AHBR (ω=0.5) in cases of different distortions of queries.

Since the above results are based on the standard COIL-100 dataset orientation, noise was artificially added to the queries. To evaluate the methods also with real IMU data, we show the results over the SUP-16 database in Figure 7.

When testing with the SMO dataset, similarly to the previous ones, the increase in the number of query images $\left(N^{q}\right)$ results in a significant increase in hit-rate (from +4.8 to +20.4%) while the effect of various backgrounds makes a difference from 2.4 to 15.6% (see Table 1). It is interesting, but not surprising, that, while the hit-rate is higher for uniform backgrounds, the improvement is greater for the textured cases. In [7], there are data only for four query cases with about 80% success. Note that, while the two test cases are not comparable from every aspect, all of our data (spreading between 80% and 98%, shown in bold) reach or overtake the performance of cases described in [7] at $N^{q}=4$ (despite the significant noise we applied on queries).

### 6.3. Active Vision for Retrieval

AHBR is a straightforward extension of HBR: since the evaluation of the weak classifiers is independent and continuous, the direction of a new viewpoint can be arbitrarily selected. In the presented experiments, the first two queries were searched by the HBR algorithm and resulted in a small set of candidates that were evaluated by AHBR and proposed a new position. There is a high probability that this set of initial selections contains the right candidate. In Figure 6, the data lines labeled as “Best 10” are the hit-rates counted so that the right candidate is within the 10 best elements after the evaluation of Equation (4). This curve is 5–14% above the best result of HBR, meaning that HBR is close to the optimal solution many times, but the randomly selected views are not satisfactory enough for an optimal decision. Through a better selection of views, we might be able to increase the discriminative power of the method.

Figure 6 contains the hit-rates for AHBR, which are, albeit below the “Best 10” as expected, significantly better than those for HBR. Data can also be interpreted in a way such that, after four queries of the active method, the results are as good or even better than the results from HBR at $N^{q}=8$. Therefore, to achieve the same accuracy, less observation is needed. Naturally, orientation measurements were loaded with noise, and all query images were distorted in the tests, where $N^{\tilde{M}}=4$.

### 6.4. Running Time and Memory Requirements

To estimate the complexity of the whole retrieval mechanism, we implemented the algorithms in Java and ran them on a tablet hosting Android 4.2.2 Jelly Bean. Since the algorithms are composed of different parts (feature extraction, indexing, and comparisons), we believe that direct time measurements reflect the complexity and the applicability better than does big O notation. The tablet was equipped with 1 GB of RAM and an ARM Cortex A9 Dual-Core 1.5 GHz Processor. Using this modest hardware, not utilizing parallelism (using a single core for our algorithms) and not having GPU functions, we were still able to achieve 1 FPS.

The memory requirements of the used CEDD vectors and the orientation data are very low. Even assuming that all 72 views of objects are stored, as originally given by the COIL-100 dataset, less than 1.5 MB is satisfactory for the storage of the models of 100 objects.

## 7. Discussion and Conclusions

Based on the large number of papers on multiple-view and video-based object retrieval, we knew that multiple observations would be more efficient than single image recognition. Here, we solve the multiple-view recognition problem with an approach where the task is to combine weak classifiers to obtain a robust recognition technique. As shown above, this is not merely as simple as obtaining visual information of the objects; the utilization of the orientation information of models and queries can also increase hit-rate (from about 2 to 5% in our examples). Moreover, moving to a new viewpoint that reduces the expected uncertainty of the identity of the object, rather than a simple one-way or random sequence of observations, can increase the hit-rates much more (in previous tests, this increase could reach 13%). Naturally, there are objects that are rotationally invariant, and the estimation of the viewing position is unreliable for them, but their unvarying views can give useful hints for recognition.

A great advantage of the Hough framework is that it can be used to create a technique where several optical descriptors and orientation information can be combined efficiently. Since the evaluation of the independent queries is relatively fast and is a continuous process, active perception can be easily carried out. Moreover, being data-driven is a great advantage that DNNs do not have: adding new objects to the system does not require retraining, and the small size KD-tree database can easily be extended or replaced. There are only a few parameters of the proposed method (the value of ω and the length of the independent retrieval lists), and we found the optimal settings easily via manual tuning.

We should also mention that the proposed methods of 3D object retrieval can be extended for object recognition and can be easily combined with tracking (for such extensions, we refer the readers to [9]).

Energy efficiency and the cost of intelligent sensors has been an important aspect for decades. To maximize the information collected by optical sensors at minimal cost, active vision approaches are necessary, as IMUs can add valuable information. The benefits of such solutions become very clear when the system has minimal computational power and memory and when only a limited amount of operation time is available (such as in the case of UAVs).

In this article, we have shown that object specific retrieval can be achieved with a combination of weak classifiers in the Hough framework, within which the fusion of optical and orientation information is also effective. As shown by experiments and data analysis, the proposed method is robust to noise, is fast, needs very little memory and few computation resources, and can implement active vision to increase retrieval performance in lightweight sensor nodes. 

## Figures and Tables

**Table 1:** Average hit-rate of the HBR method on the different variations in the SOM dataset.

# Query Views	Dataset Variations					
	DF-UB	DF-TB	MB-UB	MB-TB	GN-UB	GN-TB
2	94.4%	78.8%	84.8%	69.2%	82.4%	69.2%
4	98%	90.4%	93.6%	80%	89.6%	80.4%
6	98.4%	94.4%	95.6%	85.6%	92.4%	86%
8	99.2%	96.8%	96%	88.8%	93.6%	89.6%

## Abbreviations

The following abbreviations are used in this manuscript:

AHBR	Active-Hough-Based Retrieval
BoVW	Bag of Visual Words
CEDD	Color and Edge Directivity Descriptor
CNN	Convolutional Neural Network
DF	Distortion Free
DNN	Deep Neural Network
FAST	Features from Accelerated Segment Test
FPS	Frames per Second
GN	Gaussian Noise
GPU	Graphics Processing Unit
HBR	Hough-Based Retrieval
HSV	Hue Saturation Value
IMU	Inertial Measurement Unit
MB	Motion Blur
MI	Mutual Information
MPEG	Moving Picture Experts Group
UAV	Unmanned Aerial Vehicle
UB	Uniform Background
RGB-D	Red Green Blue—Depth
RMSE	Root Mean Square Error
SIFT	Scale Invariant Feature Transform
SLAM	Simultaneous Localization and Mapping
SURF	Speeded Up Robust Features
TB	Textured Background
